# Keratinocytes Regulate the Threshold of Inflammation by Inhibiting T Cell Effector Functions

**DOI:** 10.3390/cells10071606

**Published:** 2021-06-26

**Authors:** Peter Seiringer, Stefanie Eyerich, Kilian Eyerich, Daniela Dittlein, Anna Caroline Pilz, Emanuele Scala, Johannes Ring, Heidrun Behrendt, Andrea Cavani, Claudia Traidl-Hoffmann

**Affiliations:** 1 Division of Dermatology and Venerology, Department of Medicine Solna and Center for Molecular Medicine, Karolinska Institutet, 17176 Stockholm, Sweden; kilian.eyerich@ki.se (K.E.); emanuele.scala@ki.se (E.S.); 2 ZAUM—Center of Allergy and Environment, Technical University and Helmholtz Center Munich, 80802 Munich, Germany; caroline.pilz@tum.de (A.C.P.); heidrunbehrendt@web.de (H.B.); 3 Chair and Institute of Environmental Medicine, UNIKA-T, Technical University of Munich and Helmholtz Center Munich, 86156 Augsburg, Germany; daniela.dittlein@tum.de (D.D.); claudia.traidl-hoffmann@tum.de (C.T.-H.); 4 Department of Dermatology and Allergy, Technical University of Munich, 80802 Munich, Germany; johannes.ring@tum.de; 5 Scientific Coordination Unit, National Institute for Health, Migration and Poverty (INMP-NIHMP), 00153 Rome, Italy; andrea.cavani@inmp.it

**Keywords:** keratinocytes, T cells, T cell effector functions, skin immune homeostasis, skin barrier

## Abstract

Whilst the importance of keratinocytes as a first-line defense has been widely investigated, little is known about their interactions with non-resident immune cells. In this study, the impact of human keratinocytes on T cell effector functions was analyzed in an antigen-specific in vitro model of allergic contact dermatitis (ACD) to nickel sulfate. Keratinocytes partially inhibited T cell proliferation and cytokine production. This effect was dependent on the keratinocyte/T cell ratio and was partially reversible by increasing the number of autologous dendritic cells. The inhibition of T cell proliferation by keratinocytes was independent of the T cell subtype and antigen presentation by different professional antigen-presenting cells. Autologous and heterologous keratinocytes showed comparable effects, while the fixation of keratinocytes with paraformaldehyde abrogated the immunosuppressive effect. The separation of keratinocytes and T cells by a transwell chamber, as well as a cell-free keratinocyte supernatant, inhibited T cell effector functions to the same amount as directly co-cultured keratinocytes, thus proving that soluble factor/s account for the observed suppressive effects. In conclusion, keratinocytes critically control the threshold of inflammatory processes in the skin by inhibiting T cell proliferation and cytokine production.

## 1. Introduction

Barrier organs, such as skin, airway and gut epithelia, represent the first-line defense of the organism. To protect the inside of the body from foreign and potentially harmful substances, the skin exhibits various protective defense mechanisms, such as a mechanical barrier formed through tight junctions between keratinocytes; a chemical barrier that consists of an acidic pH; the production of defensins and other antimicrobial peptides (AMPs), proteases and cytokines; and the expression of pathogen-related receptors (PRRs), such as toll-like receptors (TLRs) and nucleotide-binding oligomerization domain (NOD)-like receptors [1,2].

Epithelial surfaces are a non-sterile environment with a local microbiome. Because of the non-adaptable receptor repertoire, innate immune mechanisms are not able to discriminate between pathogenic and non-pathogenic microorganisms. Therefore, and to sustain organ physiology, immune reactions in epithelia have to be regulated to achieve a peaceful coexistence with non-pathogen bacteria rather than sterility. This inflammatory regulation is organ-specific and depends on the threshold of activation of the PRR, the distinct expression pattern of PRRs and their regulators in the epithelium and the unique defense capacity of the organ [3]. In the skin, innate lymphoid cells (ILCs) play a crucial role in maintaining tissue homeostasis [4]. A dysregulation of ILCs and other players of innate immunity in the skin can cause psoriasis, atopic eczema and other (auto)inflammatory disorders [5,6]. For a long time, keratinocytes have been regarded as immunologically inert bystander cells. However, keratinocytes have the ability to respond to a variety of exogenous and endogenous stimuli and are important, active participants in skin immune responses. Besides their major role in the first-line defense and production of AMPs [7], keratinocytes also influence other immune cells by secreting T cell-modulating cytokines, such as IL-12 and IL-18 [8,9]; by releasing several chemokines that induce the migration of immune cells into the skin [10]; and expressing surface molecules, such as HLA class I and ICAM-1. This, in turn, could facilitate tight interactions between keratinocytes and immune cells [11]. Furthermore, keratinocytes directly interact with dendritic cells (DCs) via the anti-microbial peptide LL37 that prevents the activation of DCs [12]. In addition, DCs that have been stimulated with epithelial cells or a supernatant of epithelial cell cultures tend to induce IL-10 production and a regulatory phenotype in T cells [13,14,15,16]. Recently, it has been shown that a loss of de novo synthesis of glucocorticoids in murine keratinocytes exacerbates psoriasiform inflammation and contact hypersensitivity and is accompanied by decreased regulatory T cells in mice [17]. On the other hand, keratinocytes can also have an activating effect on T cells. Staphylococcal aureus enterotoxin B (SEB)-loaded immortalized human HaCaT keratinocytes are capable of inducing T cell proliferation without direct contact, likely through exosomes [18]. Exosomes also seem to play a central role in the communication between keratinocytes and other skin-homing cells [19]. Furthermore, keratinocytes can enhance the T helper (Th)1 and Th17 polarization of naïve T cells through direct cell contact and costimulatory signaling via CD58/CD2 and CD54/LFA-1, independent of the presence of DCs [20]. Despite these findings, still little is known about the influence of keratinocytes on T cell activity. So far, direct interactions between keratinocytes and T cells have been characterized mainly by emphasizing T cell effects on keratinocytes and not vice versa.

In this study, we investigated a possible role of the microenvironment on the development of inflammatory processes in the skin. To analyze the discourse of keratinocytes and T cells, we developed an antigen-specific in vitro model for a common inflammatory skin disease—allergic contact dermatitis (ACD) to nickel sulfate. Nickel sulfate is the most common contact allergen worldwide and has been extensively investigated [21]. As nickel sulfate occurs ubiquitously, skin exposure from cosmetics, household products and metallic items, such as watches or belt buckles, is almost inevitable [22]. The pathogenesis of ACD is characterized by the activation of innate and adaptive immune mechanisms [22]. Key events are the apoptosis of resident keratinocytes induced by skin infiltrating CD8+ and CD4+ nickel-specific T effector cells [23,24], resulting in the loss of cell integrity leading to spongiosis and the clinical symptoms of eczema.

Herein, we show that keratinocytes impede T cell effector functions, such as nickel-specific proliferation and cytokine production. This effect was dose-dependent and mediated by yet unidentified soluble factor/s. Thus, our results emphasize that keratinocytes exhibit an immunologically active part of the skin microenvironment and contribute to skin homeostasis by regulating the threshold of inflammation and protecting the skin against unnecessary and overwhelming immune responses.

## 2. Materials and Methods

### 2.1. Study Population

Nickel-sensitized patients (n = 6) with positive patch test to NiSO_4_ and no further IgE sensitization (total IgE ≤ 20 kU/L, RAST to common allergens negative) were included in the study. Before blood was taken, each patient gave their informed consent. The study was approved by the ethical committee of the Technical University of Munich.

### 2.2. Culture of Primary Human Keratinocytes

Epidermal sheets were obtained from the roof of suction blisters raised on normal skin on the forearms of donors and disaggregated to a single-cell suspension using 0.05% trypsin (Invitrogen, Paisley, Scotland). Keratinocyte primary cultures were established by seeding epidermal cells on a feeder layer of mitomycin-treated 3T3/J2 fibroblasts and cultured in modified Green’s medium as described previously [23]. At 70–80% confluence, keratinocytes were detached with 0.05% trypsin, aliquoted and cryopreserved in liquid nitrogen. Only second-passage keratinocytes were used in co-culture experiments.

Where indicated, keratinocytes were pretreated with 500 U/mL IFN-γ (R&D, Wiesbaden, Germany) for 6 h and cultured for further 24 h in keratinocyte medium without hydrocortisone or fixed with 2% paraformaldehyde (PFA) (15 min, 37 °C). After both conditions, keratinocytes were extensively washed and used in co-cultures afterwards.

### 2.3. Generation of T Cell Clones

Nickel-specific T cell clones were isolated as described previously [23]. Briefly, PBMC from nickel-sensitized patients (n = 6) were isolated and left to adhere in Petri dishes. The non-adherent fraction was separated in CD4^+^ and CD8^+^ cells by immunomagnetic labeling with bead-coupled antibodies (MACS, Miltenyi Biotech, Bergisch Gladbach, Germany). To enrich nickel-specific T cells, T cell lines were established and cloned by limiting dilution (0.6 cells/well in 96-well U-bottom microplates) in RPMI 1640 supplemented with 2 mM L-glutamine, 1 mM sodium pyruvate, 1% nonessential aminoacids, 0.05 mM 2-mercaptoethanol, 100 U/mL penicillin, 100 mg/mL streptomycin and 5% human serum (all from Invitrogen, Paisley, Scotland) (RPMI complete) in the presence of mitomycin (Sigma, München, Germany) treated PBMC, 30 U/mL IL-2 (Novartis, Basel, Switzerland) and 1% PHA (Sigma, München, Germany). Clones were grown by adding IL-2 (30 U/mL) twice a week and periodically restimulated with 1% PHA in the presence of mitomycin-treated PBMC or plate-coated anti-CD3 (1 mg/mL) and soluble anti-CD28 (1 mg/mL) (both BD Biosciences, Heidelberg, Germany). The nickel reactivity of both T cell lines and clones were assayed by the incorporation of radioactive-labeled ^3^H-thymidin. The cytokine pattern of each clone was evaluated in supernatants using commercially available ELISA kits for IFN-γ, IL-4 and IL-10 (BD Biosciences, Heidelberg, Germany) 48 h after activation with autologous antigen-presenting cells (APCs) and antigen. The T cell subtype was defined on the basis of Sager et al. [25] and Sebastiani et al. [26]. T cell clones were characterized as follows: Th0 clones secrete IFN-γ, IL-4 and IL-10 in equal amounts; Th1 clones produce more than 80% IFN-γ, no IL-4 and less than 20% IL-10; Th2 clones secrete more than 60% IL-4 and less than 20% IFN-γ; and Tr1 clones produce more than 65% IL-10.

### 2.4. Generation of Monocyte-Derived DCs

Monocyte-derived DCs were prepared from the peripheral blood of nickel-sensitized donors, as described recently [27]. In brief, adherent PBMC (90% pure CD14^+^ cells) were cultured in RPMI 1640 supplemented with 2 mM L-glutamine, 0.5 mM 2-mercaptoethanol, 20 µg/mL gentamycin (all from Invitrogen, Paisley, Scottland) 10% FCS, 50 U/mL human rGM-CSF and 50 U/mL human rIL-4 (Immunotools, Friesoythe, Germany) (complete DC medium) at 37 °C under 5% CO_2_. At day 5, cells (95% CD1a^+^, CD14^−^) were harvested and used in co-culture experiments.

### 2.5. Keratinocyte/T Cell Co-Culture

Co-culture of autologous T cells, keratinocytes and DCs was performed in RPMI complete and 96-well flat-bottom microplates (Nunc, Roskilde, Denmark). Nickel sulfate (20 µg/mL) (Sigma, München, Germany) was used as antigen. Specific T cell proliferation was measured after 48 h by the incorporation of radioactive-labeled ^3^H-thymidin (GE Healthcare, München, Germany). A proliferation index (PI), defined as the ratio of T cell proliferation with and without antigens, greater than 2 was regarded as specific proliferation. T cell cytokine production was evaluated in cell-free supernatants by ELISA (BD Biosciences, Heidelberg, Germany).

Co-culture settings were varied by using the supernatants of keratinocyte cultures instead of keratinocytes and by the separation of T cells and keratinocytes via a transwell chamber (Nunc, Roskilde, Denmark).

### 2.6. Basic Multi Epitope Ligand Cartography (MELC) Set-Up

MELC robot technology [28,29] was applied by using a toponome imaging cycler. Briefly, a slide with a skin section was positioned onto the stage of an inverted fluorescence microscope (Leica DM IRE2; 20× air objective lens), equipped with fluorescence filters for FITC and phycoerythrin. Through a robotic process of on/off-pipetting, the specimen was incubated with fluorophore-labeled tags (validated MELC library of 49 tags, mostly antibodies) and wash solutions under temperature and time control. The phase contrast view and fluorescence signals were recorded by a cooled CCD camera (Apogee KX4, 2048 × 2048 pixels), followed by soft bleaching. The recording of all image data and the coordination of all system components were controlled by software from meltec GmbH & Co. KG. All these processes (tag incubation/fluorescence detection/soft bleaching) were part of a fully automated cycle repeated for any number of tag binding sites (epitopes). As described in detail earlier [29], image preprocessing mainly comprised (1) a pixel-precise overlay of fluorescence images, (2) image correction for background and illumination faults and (3) the exclusion of invalid pixels that were not part of the information associated with the biological specimen (e.g., section artifacts).

### 2.7. Statistical Analysis

Statistical analysis was performed by the GraphPad Prism software. If not indicated otherwise, results were analyzed using the Wilcoxon signed rank test orone-way ANOVA with Tukey correction for multiple testing. Statistically significant differences were defined as *: *p* < 0.05; **: *p* < 0.01; ***: *p* < 0.001; ****: *p* < 0.0001.

## 3. Results

### 3.1. Close Morphological Interactions between Keratinocytes and T Cells in an In Vitro Model of Allergic Contact Dermatitis

**Figure 1:**
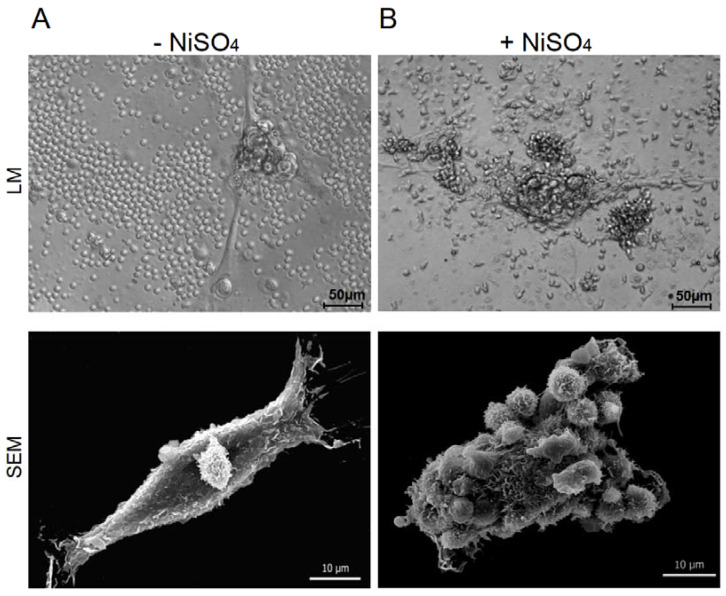
Keratinocytes and nickel-specific T cell clones interact in an autologous in vitro model of allergic contact dermatitis. Nickel-specific T cell clones were incubated together with autologous DCs and keratinocytes in the absence (**A**) or presence (**B**) of their specific antigen. The interaction of T cells and keratinocytes was monitored in light microscopy (LM) and scanning electron microscopy (SEM).

Keratinocytes, nickel-specific T cells and autologous DCs were incubated in the presence or absence of nickel sulfate. The interplay between T cells and keratinocytes was followed by light microscopy and scanning electron microscopy (SEM) (Figure 1). The activation of T cells with their specific antigen (Figure 1B) resulted in close morphological interactions between T cells and keratinocytes, while resting T cells did not engage with keratinocytes (Figure 1A). The close interactions of activated T cells were stable and even persisted after the extensive washing steps for sample preparation in SEM (Figure 1, lower panel), thus suggesting the set-up of a synapse between antigen-specific T cells and keratinocytes. These results show that the presented in vitro model of ACD closely resembles the in vivo situation with skin-infiltrating T cells interacting with resident cells.

### 3.2. Keratinocytes Suppress Antigen-Specific Proliferation and Cytokine Production of T Cell Clones

**Figure 2:**
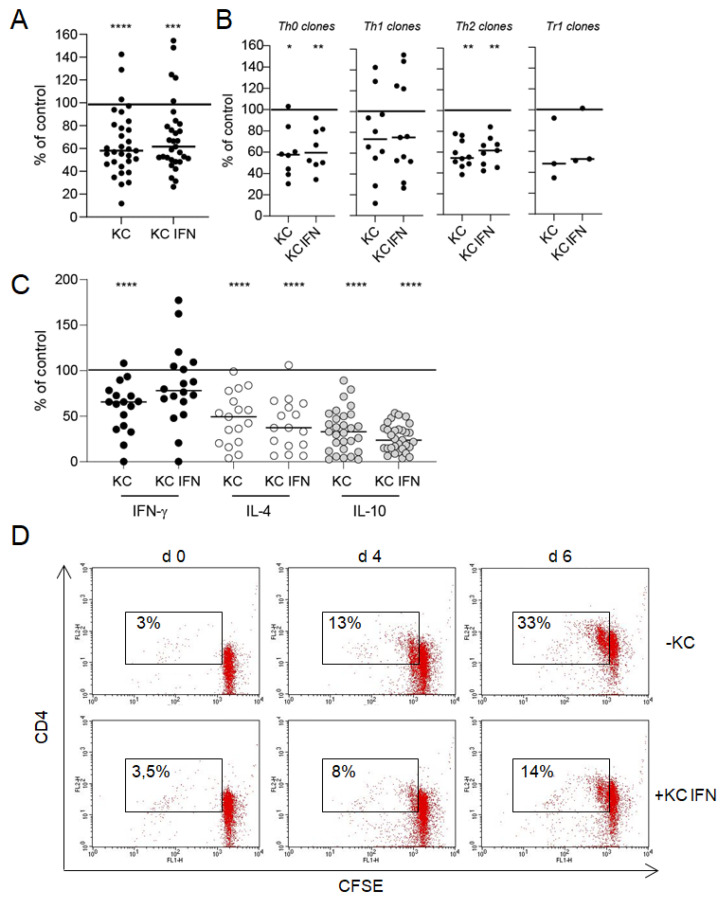
Interaction between keratinocytes and T cells results in an inhibition of T cell effector functions. Keratinocytes (KC) or IFN-γ prestimulated keratinocytes (KC IFN), autologous nickel-specific T cell clones and DCs were incubated in a ratio of 10:100:1 in the presence of nickel sulfate. After 48 h, nickel-specific T cell proliferation was measured by 3H-thymidine incorporation (**A**,**B**), and the production of cytokines was detected in a cell-free co-culture supernatants by ELISA (**C**). Each data point represents one T cell clone with an average of at least three experiments (T cell clones pooled, panel (**A**); T cell clone subsets, panel (**B**)). The line displays the median of all clones investigated. Shown is the percentage change of T cell proliferation and cytokine production compared to the control culture without keratinocytes (normalized to 100%). The inhibitory effect of keratinocytes on T cell proliferation could also be observed in a flow cytometric approach with CFSE-labeled T cell clones (**D**). The number indicates the CD4+, proliferating T cells. ((**A**–**C**) clones = 32, n = 105, (**D**) one representative clone in co-culture with IFN-γ prestimulated keratinocytes; *: *p* < 0.05; **: *p* < 0.01; ***: *p* < 0.001; ****: *p* < 0.0001).

To further characterize the microscopically observed interactions between keratinocytes and T cells, we investigated T cell effector functions, such as proliferation and cytokine production in a co-culture system. Keratinocytes significantly inhibited nickel-specific T cell proliferation compared to the control culture (T cell clones with APCs and nickel sulfate without keratinocytes, set to 100%) (Figure 2A,B). The prestimulation of keratinocytes with IFN-γ did not alter the inhibitory impact on T cell proliferation. Only a few Th1 T cell clones were not affected (proliferation 100% = 11956 ± 957 cpm; proliferation KC = 8155 ± 973 cpm; proliferation KC IFN = 8333 ± 901 cpm). The inhibitory effect of keratinocytes on T cell proliferation was also observed in co-culture experiments with CFSE-labeled T cells (Figure 2D).

Cytokine production in activated T cells was also inhibited by keratinocytes (IFN-γ 100% = 9802 +/− 2551 pg/mL; IL-4 100% = 2197 +/− 525 pg/mL; IL-10 100% = 834 +/− 204 pg/mL) (Figure 2C). IL-10 suppression was most prominent and showed the highest significance, followed by IL-4 and IFN-γ. Again, IFN-γ prestimulation of keratinocytes did not alter the inhibitory effect on T cell cytokine production. However, the suppression of IL-4 and IL-10 was higher in co-cultures with IFN-γ prestimulated keratinocytes without reaching statistical significance. Notably, the modification of T cell effector functions was independent of the T cell subtype (Figure 2). To rule out that the differentiation status of keratinocytes influences their capacity to inhibit T cell proliferation and T cell effector functions, we performed experiments with keratinocytes that have been cultured under differentiating conditions (1.2 mM CaCl2) for 24 h before usage in co-culture experiments with T cells (Supplementary Figure S1). Keratinocyte differentiation does not impact the capacity of keratinocytes to inhibit T cell proliferation and cytokine production.

### 3.3. Keratinocytes Inhibit T Cell Effector Functions in a Dose-Dependent Manner

**Figure 3:**
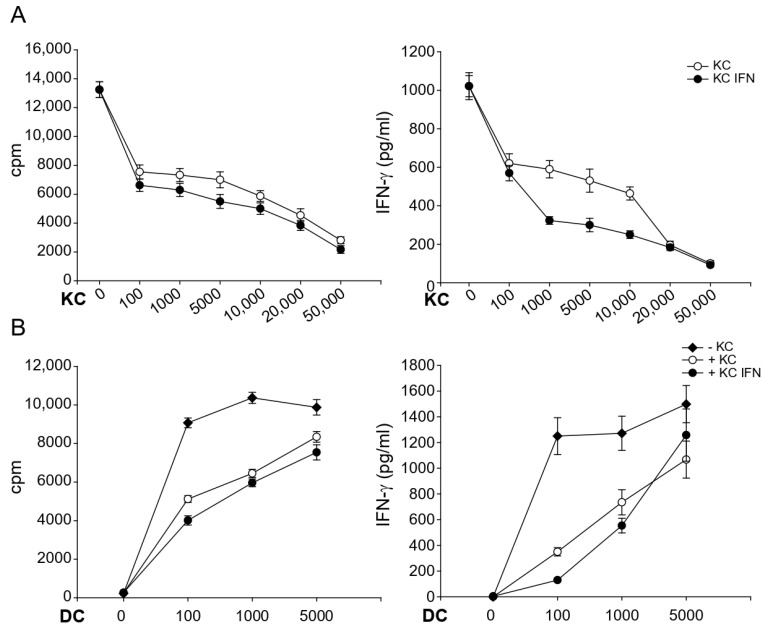
The modulatory capacity of keratinocytes is dose-dependent and dependent on the strength of antigen presentation. Nickel-specific T cell clones (1 × 10^5^) were incubated together with nickel sulfate, autologous DCs (1 × 10^3^) and different numbers of unstimulated keratinocytes (**A**) or with 1 × 10^4^ keratinocytes and different numbers of DCs (**B**). Proliferation and cytokine production was measured after 48 h by ^3^H-thymidine and ELISA, respectively. Shown are the representative results of one nickel-specific Th1 clone (n = 3).

Nickel-specific T cells clones were activated by autologous DCs in the presence of increasing numbers of autologous keratinocytes. As little as 1 × 10^2^ keratinocytes had significant suppressive effects on proliferation and cytokine production in 1 × 10^5^ activated T cells. However, suppression augmented with increasing numbers of keratinocytes in a dose-dependent manner, being strongest in a 1:2 ratio and more pronounced with IFN-γ prestimulated keratinocytes. The results of one representative nickel-specific Th1 clone are shown in Figure 3A.

To evaluate whether the strength of T cell activation has any impact on the observed effects, we varied the numbers of DCs (Figure 3B). While keratinocytes strongly suppressed T cell effector functions in the presence of suboptimal numbers of DC (between 1 × 10^2^ and 1 × 10^3^), suppression was abrogated almost completely in the presence of 5 × 10^3^ DCs. These results indicate that keratinocytes continuously produce T cell suppressive factors. However, very strong T cell activation can partially abrogate this inhibition.

### 3.4. Keratinocytes Inhibit T Cell Effector Functions by Secreting Suppressive Soluble Factors

**Figure 4:**
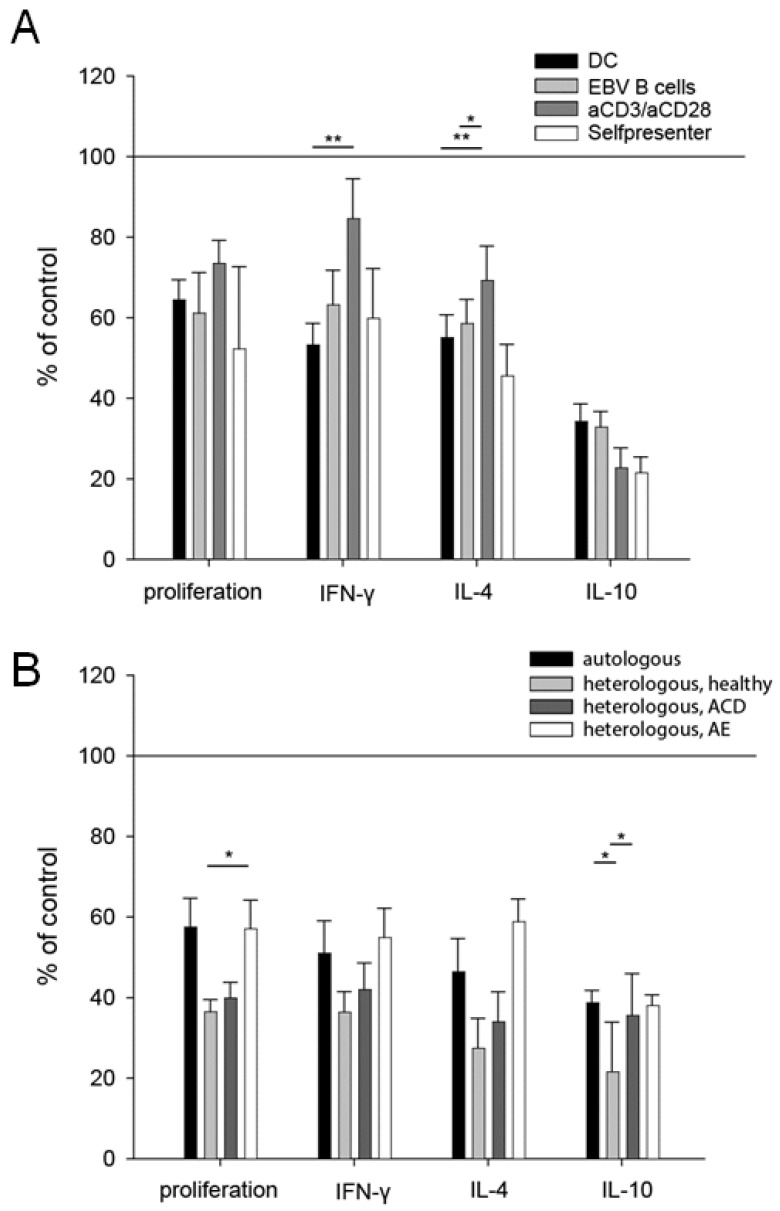
The inhibitory capacity of keratinocytes is independent of the antigen presentation and HLA-TCR interactions. Nickel-specific T cell clones were co-cultured with autologous keratinocytes, nickel sulfate and different types of APCs (dendritic cells (DC), Epstein–Barr-virus-transfected B cells (EBV B cells)), TCR stimuli (anti-CD3/anti-CD28 antibodies) or in the absence of professional APCs as self-presenter cells (self) (**A**). Comparison of the co-culture of nickel-specific T cells, DCs, nickel sulfate and autologous keratinocytes to the co-culture with heterologous keratinocytes (**B**). The heterologous keratinocytes were obtained from a healthy volunteer, a patient with ACD to nickel and one patient with atopic eczema/dermatitis (AE). All conditions showed significant differences, compared to the control condition (set to 100%) (*p* < 0.05). Results were analyzed using one-way ANOVA with Tukey correction for multiple testing. ((**A**) DC clones = 13, n = 50; EBV clones 20, n = 53; aCD3/aCD28 clones 8, n = 25; self-presenter clones 8, n = 33; (**B**) autologous n = 35; heterologous healthy n = 10; heterologous ACD n = 22; heterologous AE n = 8); *: *p* < 0.05; **: *p* < 0.01).

To elucidate the underlying mechanisms of the cross-talk between keratinocytes and T cells, the co-culture settings were varied. The modulation of T cells by keratinocytes was independent of APCs, as different types of APCs (monocyte-derived DC and EBV-transformed B cells) and the APC-independent activation of T cells (nickel-specific self-presenter T cells which proliferate independently of professional APCs [30] and TCR stimulation with antiCD3/antiCD28) resulted in comparable inhibition of both T cell proliferation and cytokine production (Figure 4A). Furthermore, the observed inhibition was not restricted to MHC-dependent mechanisms, because heterologous keratinocytes of healthy, atopic, nickel-sensitized and atopic eczema patients were able to inhibit the effector functions of nickel-specific T cell clones as much as autologous keratinocytes (Figure 4B).

**Figure 5:**
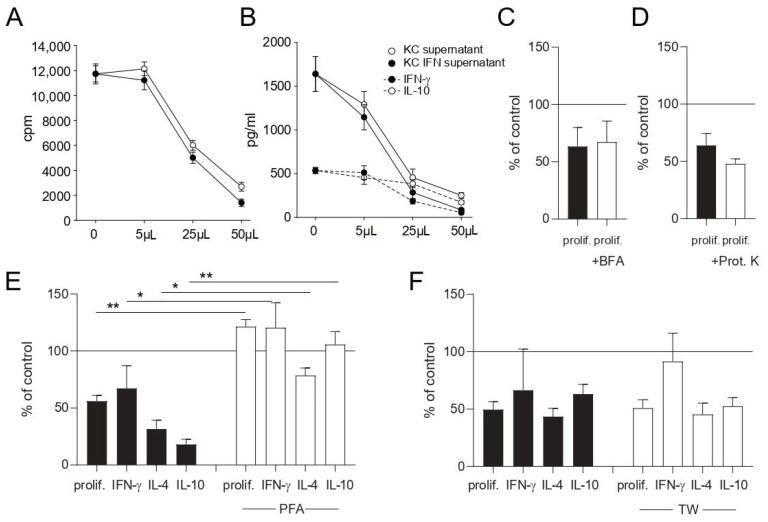
Keratinocytes inhibit T cell effector functions through the production of soluble factor/s. Cell-free culture supernatants of keratinocyte- or IFN-γ-prestimulated keratinocyte cultures (KC IFN) were obtained and used in different volumes in co-culture experiments. Proliferation (**A**) and cytokine production (**B**) were measured after 48 h by 3H-thymidine incorporation and ELISA of co-culture supernatants, respectively. Unstimulated keratinocytes were fixed with PFA (**E**) or treated with brefeldin A (BFA) to block the Golgi apparatus (**C**) prior to co-culture with nickel-specific T cell clones and dendritic cells. Supernatants of unstimulated keratinocytes were treated for 30 min with proteinase K (Prot. K) (**D**) prior to co-culture with nickel-specific T cell clones and dendritic cells. Keratinocytes were separated from nickel-specific T cells and dendritic cells via a transwell chamber (TW) (**F**). To rule out any effects from the keratinocyte medium, the medium was added to every control stimulation in the same volume as was used for the keratinocyte supernatant experiments. ((**A**) results of one representative nickel-specific Th1 clone; (**E**) clones = 8, n = 16; (**C**,**D**) clones = 4, n = 8; (**F**) clones = 5, n = 14; *: *p* < 0.05; **: *p* < 0.01).

In contrast, the fixation of keratinocytes with PFA before adding them in the co-culture system abrogated this inhibitory effect on T cell proliferation and cytokine production (Figure 5E). In order to distinguish between soluble factors and cell-to-cell contact-dependent mechanisms, T cells and keratinocytes were separated by a transwell chamber. There was no significant difference between transwell-separated and directly co-cultured keratinocytes concerning the inhibition of proliferation and cytokine production in nickel-specific T cell clones (Figure 5F). Cell-free keratinocyte culture supernatants also suppressed T cell proliferation (Figure 5A) and cytokine production (Figure 5B) in a dose-dependent manner, thus proving that keratinocytes modulate T cell effector functions by secreting suppressive soluble factor/s. To investigate whether proteins account for the effect, unstimulated keratinocytes were treated with brefeldin A (BFA) to block the Golgi apparatus prior to co-culture with nickel-specific T cell clones and dendritic cells (Figure 5C). Additionally, supernatants of unstimulated keratinocytes were treated with proteinase K (Figure 5D) prior to co-culture. No significant changes between untreated and treated cells were observed, thus suggesting a protein-independent effect.

### 3.5. T Cells Do Not Express Proliferation Markers in the Skin In Vivo

**Figure 6:**
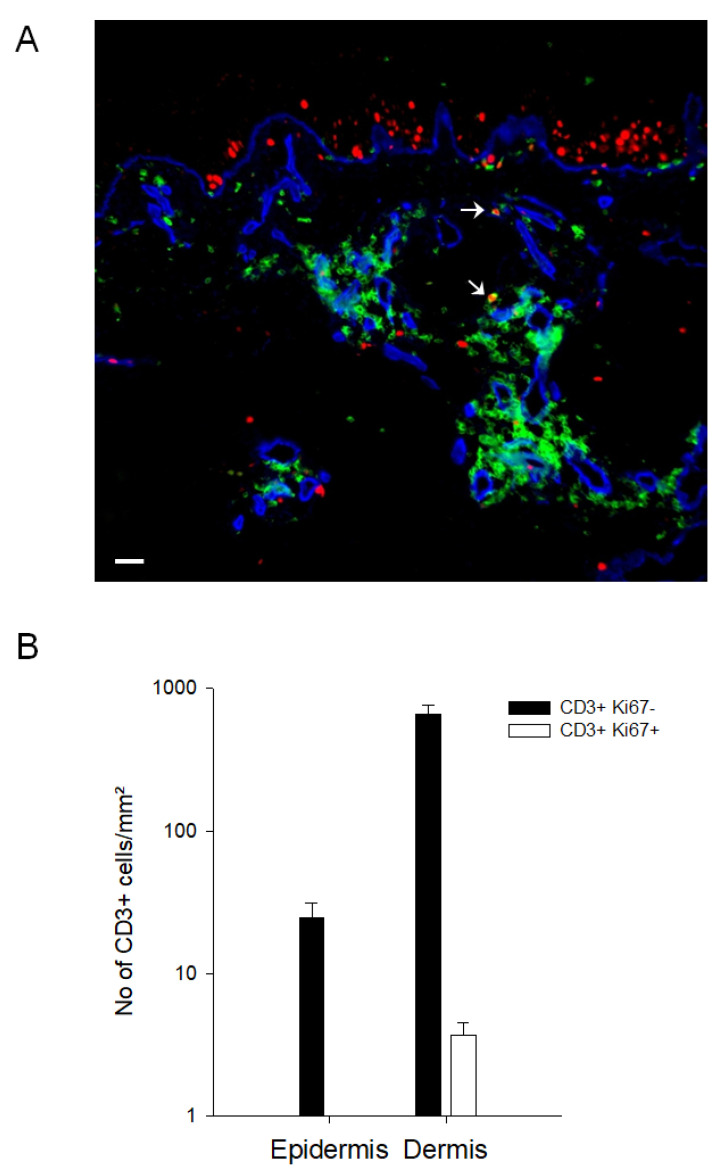
Skin-infiltrating T cells do not proliferate in the skin micromilieu in vivo. Biopsies from patients (n = 6) with a positive nickel-patch test were obtained and analyzed for the expression of Ki67 (red), CD3 (green) and CollagenIV (blue) with the MELC technology. In (**A**), one representative RGB overlay is shown. Arrows mark CD3/Ki67 double positive T cells. Scale bar indicates 20 µm. (**B**) CD3+ Ki67- and CD3+ Ki67+ double positive cells were counted in the epidermis and dermis (n = 6).

To investigate the inhibitory capacity of keratinocytes on T cell proliferation in vivo, skin biopsies from nickel-patch tests (n = 6) were analyzed by the MELC technique [28,29]. Figure 6 shows a post-processing overlay of three individual fluorescent stainings of the same section: Ki67 (a nuclear marker for cellular proliferation), CD3 and CollagenIV. Proliferating, Ki67+ keratinocytes are situated close to the basal membrane, whereas CD3+ T cells are mainly located in the dermal compartment. Only few CD3+ T cells show co-expression with the proliferation marker Ki67 (Figure 6).

## 4. Discussion

Whilst the importance of keratinocytes as first-line defense has been widely investigated, little is known about if and how they influence non-resident immune cells. In this study, we show that keratinocytes release soluble factors that partially inhibit T cell effector functions such as proliferation and cytokine production. Thereby, keratinocytes are critically involved in determining the threshold of inflammatory processes in the skin.

A prerequisite for analysis of keratinocyte/T cell interactions in the human system is an in vitro model which mimics the immune response in the skin. By isolating nickel-specific T cell clones, keratinocytes and APCs from patients with a positive nickel-patch test, we established an autologous, antigen-specific in vitro model of ACD. Morphological interactions between keratinocytes and T cells, T cell proliferation and T cell cytokine production were analyzed in this model.

Once activated by their cognate antigen, T cells establish close morphological interactions with autologous keratinocytes. These interactions are based on a firm adhesion of both cells’ types—presumably forming a synapse for cell–cell interactions, such as the induction of apoptosis [23,31,32,33].

Beyond this well-known cell–cell contact-dependent crosstalk, we observed a modulatory activity in keratinocytes, concerning T cell effector functions that are mediated by soluble factor/s. Thus, we extend previous data that indicate that keratinocytes induce a proliferative unresponsiveness in T cells [34,35]. This effect is dependent on the keratinocyte/T cell ratio and is partially revertible by increasing numbers of DCs, thus pointing to the fact that keratinocytes might set a threshold of inflammation, but, during an ongoing immune reaction with full activation of T cells, this effect is overwhelmed by further infiltrating DCs and T cells [36].

What soluble factor/s account for the inhibition of T cell effector functions remains to be elucidated. In order to investigate whether proteins are responsible for the effect, we treated keratinocytes with brefeldin A to block the endoplasmatic reticulum (Figure 5C) and forced the digestion of proteins by adding proteinase K in another experiment (Figure 5D). Both experiments indicated no significant changes between untreated and treated cells, suggesting a protein-independent effect. Therefore, it is likely that keratinocyte-derived lipids or metabolites mediate the observed effect. Extracellular vesicles (EV), secretory lipid bilayer membranes, are key players in the communication between skin-homing cells like keratinocytes and other skin cells [19]. They contain mRNA, miRNA, low amounts of DNA and numerous proteins and are potentially responsible for the effect. Despite the anti-inflammatory activity of keratinocyte exosomes, a subtype of EV, which has been investigated previously, a responsible factor has not yet been identified [19]. Keratinocyte-derived de novo-synthesized glucocorticoids are also potential candidates that could be responsible for the effect. In an experimental model for inflammatory skin disorders, deficiency of skin glucocorticoids led to psoriasiform skin inflammation, exacerbated contact hypersensitivity and decreased regulatory T cells [17]. On the other hand, Cai et al. recently showed that SEB-loaded HaCaT keratinocytes can induce thee proliferation of resting T cells, also likely mediated by exosomes [18]. At first sight, this seems contradictory to our results. HaCaT cells that have not been previously treated with the superantigen SEB, though, were not able to induce T cell proliferation and served as a negative control, which is in line with our experiments [18].

No significant difference in the inhibitory effect on T cellular proliferation by keratinocytes between distinct T cell subtypes was observed. Concerning the effect on cytokine production, Th2 cells were inhibited in their secretion of their effector cytokine IL-4, whereas the effector cytokine of Th1 cells, IFN-γ, was not influenced constantly. However, IL-10, which is not only a cytokine of Tr1 cells but is also produced by many other skin-infiltrating T cells upon strong activation, was inhibited throughout. This again supports the threshold theory that keratinocytes are not capable of suppressing the dominant cytokine of ACD but do dampen the general activation status of skin infiltrating T cells.

In contrast to previous studies pointing to effects of airway epithelial cells on T cells via DCs [13,14,15,37], the suppressive effects reported in this study seem to act directly on T cells. They do not depend on the type of APCs and are equally observed after specific T cell receptor stimulation with anti-CD3 and anti-CD28 antibodies, as well as in the absence of professional APCs in experiments with nickel-specific self-presenter T cell clones [30].

In order to characterize the mechanisms of keratinocytes influencing T cell effector functions, we varied our experimental co-culture settings. T cell cytotoxicity partially depends on HLA-T cell receptor (TCR) recognition [23]. In contrast, the binding of HLA and TCR is not a precondition for T cell inhibition by keratinocytes, as autologous and heterologous keratinocytes show equal effects.

Furthermore, the fixation of keratinocytes with PFA abrogated the immunosuppressive effect, suggesting that direct interactions between keratinocytes and T cells are not necessary. The separation of keratinocytes and T cells by a transwell chamber, as well as a cell-free keratinocyte supernatant, inhibit T cell effector functions equally as directly as co-cultured keratinocytes, thus proving that soluble factor/s account for the observed suppressive effects. Taking into account that the majority of invading T cells are located in the dermis without direct contact to keratinocytes, an effective modulation is only warranted by the secretion of soluble factors.

The function of regulatory cytokines in the airway has been extensively studied [38]. Notably, TGF-beta can inhibit T cell proliferation by bronchial epithelial cells [15]. Nonetheless, keratinocyte-derived TGF-β is not required to suppress skin autoimmunity and maintain skin immune homeostasis [39]. The blockage of TGF-β in our in vitro system by specific antibodies did not abrogate the suppressive effect of keratinocytes on T cell functions, thus making keratinocyte-derived TGF-β an unlikely candidate for being the responsible factor.

Keratinocytes that have been prestimulated with IFN-γ in vitro are the counterpart of keratinocytes involved in an immune reaction in vivo. IFN-γ prestimulated keratinocytes tended to more strongly suppress T cell effector functions than unstimulated cells. In line with that observation, in situ immunofluorescence stainings of nickel-patch test biopsies with MELC technology [28,29] showed that skin-infiltrating T cells mainly have a CD3+ Ki67- [40] phenotype. Although several other cell types could also potentially be involved in the effect, this suggests that T cells do not proliferate in the local skin microenvironment in vivo.

ACD inflammatory reactions are self-limited by the removal of antigens by circulating lymph, activation-induced cell death in skin-infiltrating T cells [41] and infiltrating regulatory T cells that dampen the activation and effector functions of T cells via cell–cell contact-dependent mechanisms [42] or IL-10 [26,43,44]. In addition, dermal fibroblasts have been shown to act as local immunosuppressors via the production of indoleamine 2,3-dioxygenase (IDO) [45]. Most likely, keratinocytes are also involved hand-in-hand with regulatory T cells in the limitation scenario by further inhibiting T cell effector functions and inducing repair mechanisms in injured skin areas.

In summary, our results reveal that keratinocytes are an active part of the immuno-modulatory skin microenvironment. The inhibition of T cell proliferation and cytokine production via soluble factor/s could have a strong impact on the balance between proinflammatory and tolerogenic signals during eczema and other inflammatory skin diseases.

## Data Availability

The study did not create new material and does not contain gene expression data.

## Supplementary Materials

The following are available online at https://www.mdpi.com/article/10.3390/cells10071606/s1, Figure S1: Keratinocyte differentiation does not impact the capacity of keratinocytes to inhibit T cell proliferation and cytokine production.

## References

[1] Eyerich S., Eyerich K., Traidl-Hoffmann C., Biedermann T. (2018). Cutaneous Barriers and Skin Immunity: Differentiating A Connected Network. Trends Immunol..

[2] Brandner J.M., Zorn-Kruppa M., Yoshida T., Moll I., Beck L.A., De Benedetto A. (2015). Epidermal tight junctions in health and disease. Tissue Barriers.

[3] Raz E. (2007). Organ-specific regulation of innate immunity. Nat. Immunol..

[4] Jacquelot N., Luong K., Seillet C. (2019). Physiological Regulation of Innate Lymphoid Cells. Front. Immunol..

[5] Kobayashi T., Ricardo-Gonzalez R.R., Moro K. (2020). Skin-Resident Innate Lymphoid Cells—Cutaneous Innate Guardians and Regulators. Trends Immunol..

[6] Garzorz-Stark N., Lauffer F., Krause L., Thomas J., Atenhan A., Franz R., Roenneberg S., Boehner A., Jargosch M., Batra R. (2018). Toll-like receptor 7/8 agonists stimulate plasmacytoid dendritic cells to initiate TH17-deviated acute contact dermatitis in human subjects. J. Allergy Clin. Immunol..

[7] Wang G. (2014). Human antimicrobial peptides and proteins. Pharmaceuticals.

[8] Muller G., Saloga J., Germann T., Bellinghausen I., Mohamadzadeh M., Knop J., Enk A.H. (1994). Identification and induction of human keratinocyte-derived IL-12. J. Clin. Investig..

[9] Nakanishi K., Yoshimoto T., Tsutsui H., Okamura H. (2001). Interleukin-18 regulates both Th1 and Th2 responses. Annu. Rev. Immunol..

[10] Sebastiani S., Albanesi C., De P.O., Puddu P., Cavani A., Girolomoni G. (2002). The role of chemokines in allergic contact dermatitis. Arch. Dermatol. Res..

[11] Albanesi C., Cavani A., Girolomoni G. (1998). Interferon-gamma-stimulated human keratinocytes express the genes necessary for the production of peptide-loaded MHC class II molecules. J. Investig. Dermatol..

[12] Kandler K., Shaykhiev R., Kleemann P., Klescz F., Lohoff M., Vogelmeier C., Bals R. (2006). The anti-microbial peptide LL-37 inhibits the activation of dendritic cells by TLR ligands. Int. Immunol..

[13] Pichavant M., Taront S., Jeannin P., Breuilh L., Charbonnier A.S., Spriet C., Fourneau C., Corvaia N., Heliot L., Brichet A. (2006). Impact of bronchial epithelium on dendritic cell migration and function: Modulation by the bacterial motif KpOmpA. J. Immunol..

[14] Akbari O., DeKruyff R.H., Umetsu D.T. (2001). Pulmonary dendritic cells producing IL-10 mediate tolerance induced by respiratory exposure to antigen. Nat. Immunol..

[15] Mayer A.K., Bartz H., Fey F., Schmidt L.M., Dalpke A.H. (2008). Airway epithelial cells modify immune responses by inducing an anti-inflammatory microenvironment. Eur. J. Immunol..

[16] Schulke S. (2018). Induction of Interleukin-10 Producing Dendritic Cells As a Tool to Suppress Allergen-Specific T Helper 2 Responses. Front. Immunol..

[17] Phan T.S., Schink L., Mann J., Merk V.M., Zwicky P., Mundt S., Simon D., Kulms D., Abraham S., Legler D.F. (2021). Keratinocytes control skin immune homeostasis through de novo-synthesized glucocorticoids. Sci. Adv..

[18] Cai X.W., Zhu R., Ran L., Li Y.Q., Huang K., Peng J., He W., Zhou C.L., Wang R.P. (2017). A novel noncontact communication between human keratinocytes and T cells: Exosomes derived from keratinocytes support superantigeninduced proliferation of resting T cells. Mol. Med. Rep..

[19] Nasiri G., Azarpira N., Alizadeh A., Goshtasbi S., Tayebi L. (2020). Shedding light on the role of keratinocyte-derived extracellular vesicles on skin-homing cells. Stem Cell Res. Ther..

[20] Orlik C., Deibel D., Kublbeck J., Balta E., Ganskih S., Habicht J., Niesler B., Schroder-Braunstein J., Schakel K., Wabnitz G. (2020). Keratinocytes costimulate naive human T cells via CD2: A potential target to prevent the development of proinflammatory Th1 cells in the skin. Cell. Mol. Immunol..

[21] Alinaghi F., Bennike N.H., Egeberg A., Thyssen J.P., Johansen J.D. (2019). Prevalence of contact allergy in the general population: A systematic review and meta-analysis. Contact Dermat..

[22] Ahlstrom M.G., Thyssen J.P., Wennervaldt M., Menne T., Johansen J.D. (2019). Nickel allergy and allergic contact dermatitis: A clinical review of immunology, epidemiology, exposure, and treatment. Contact Dermat..

[23] Traidl C., Sebastiani S., Albanesi C., Merk H.F., Puddu P., Girolomoni G., Cavani A. (2000). Disparate cytotoxic activity of nickel-specific CD8+ and CD4+ T cell subsets against keratinocytes. J. Immunol..

[24] Trautmann A., Akdis M., Blaser K., Akdis C.A. (2000). Role of dysregulated apoptosis in atopic dermatitis. Apoptosis.

[25] Sager N., Feldmann A., Schilling G., Kreitsch P., Neumann C. (1992). House dust mite-specific T cells in the skin of subjects with atopic dermatitis: Frequency and lymphokine profile in the allergen patch test. J. Allergy Clin. Immunol..

[26] Sebastiani S., Allavena P., Albanesi C., Nasorri F., Bianchi G., Traidl C., Sozzani S., Girolomoni G., Cavani A. (2001). Chemokine receptor expression and function in CD4+ T lymphocytes with regulatory activity. J. Immunol..

[27] Sallusto F., Lanzavecchia A. (1994). Efficient presentation of soluble antigen by cultured human dendritic cells is maintained by granulocyte/macrophage colony-stimulating factor plus interleukin 4 and downregulated by tumor necrosis factor alpha. J. Exp. Med..

[28] Schubert W. (2003). Topological proteomics, toponomics, MELK-technology. Adv. Biochem. Eng. Biotechnol..

[29] Schubert W., Bonnekoh B., Pommer A.J., Philipsen L., Bockelmann R., Malykh Y., Gollnick H., Friedenberger M., Bode M., Dress A.W. (2006). Analyzing proteome topology and function by automated multidimensional fluorescence microscopy. Nat. Biotechnol..

[30] Nasorri F., Sebastiani S., Mariani V., De Pita O., Puddu P., Girolomoni G., Cavani A. (2002). Activation of nickel-specific CD4+ T lymphocytes in the absence of professional antigen-presenting cells. J. Investig. Dermatol..

[31] Schwarz T. (2000). No eczema without keratinocyte death. J. Clin. Investig..

[32] van den Bogaard E.H., Tjabringa G.S., Joosten I., Vonk-Bergers M., van Rijssen E., Tijssen H.J., Erkens M., Schalkwijk J., Koenen H. (2014). Crosstalk between keratinocytes and T cells in a 3D microenvironment: A model to study inflammatory skin diseases. J. Investig. Dermatol..

[33] Martin G., Guerard S., Fortin M.M., Rusu D., Soucy J., Poubelle P.E., Pouliot R. (2012). Pathological crosstalk in vitro between T lymphocytes and lesional keratinocytes in psoriasis: Necessity of direct cell-to-cell contact. Lab. Investig..

[34] Gaspari A.A., Jenkins M.K., Katz S.I. (1988). Class II MHC-bearing keratinocytes induce antigen-specific unresponsiveness in hapten-specific Th1 clones. J. Immunol..

[35] Gaspari A.A., Katz S.I. (1991). Induction of in vivo hyporesponsiveness to contact allergens by hapten-modified Ia+ keratinocytes. J. Immunol..

[36] Eyerich K., Huss-Marp J., Darsow U., Wollenberg A., Forster S., Ring J., Behrendt H., Traidl-Hoffmann C. (2007). Pollen Grains Induce a Rapid and Biphasic Eczematous Immune Response in Atopic Eczema Patients. Int. Arch. Allergy Immunol..

[37] Bu T., Wang L.F., Yin Y.Q. (2020). How Do Innate Immune Cells Contribute to Airway Remodeling in COPD Progression?. Int. J. Chronic Obstr. Pulm. Dis..

[38] Branchett W.J., Lloyd C.M. (2019). Regulatory cytokine function in the respiratory tract. Mucosal Immunol..

[39] Yang Y., Zenke Y., Hirai T., Kaplan D.H. (2019). Keratinocyte-derived TGFbeta is not required to maintain skin immune homeostasis. J. Dermatol. Sci..

[40] Scholzen T., Gerdes J. (2000). The Ki-67 protein: From the known and the unknown. J. Cell. Physiol..

[41] Krammer P.H., Arnold R., Lavrik I.N. (2007). Life and death in peripheral T cells. Nat. Rev. Immunol..

[42] Cavani A., Nasorri F., Ottaviani C., Sebastiani S., De Pita O., Girolomoni G. (2003). Human CD25+ regulatory T cells maintain immune tolerance to nickel in healthy, nonallergic individuals. J. Immunol..

[43] Verhagen J., Akdis M., Traidl-Hoffmann C., Schmid-Grendelmeier P., Hijnen D., Knol E.F., Behrendt H., Blaser K., Akdis C.A. (2006). Absence of T-regulatory cell expression and function in atopic dermatitis skin. J. Allergy Clin. Immunol..

[44] Martin S.F., Rustemeyer T., Thyssen J.P. (2018). Recent advances in understanding and managing contact dermatitis. F1000Research.

[45] Ghahary A., Li Y., Tredget E.E., Kilani R.T., Iwashina T., Karami A., Lin X. (2004). Expression of indoleamine 2,3-dioxygenase in dermal fibroblasts functions as a local immunosuppressive factor. J. Investig. Dermatol..

