# Supplementary material for: Keratinocytes Regulate the Threshold of Inflammation by Inhibiting T Cell Effector Functions
Source: Cells. 2021 Jun 26;10(7):1606. doi: 10.3390/cells10071606 (PMC8306889; doi:10.3390/cells10071606)
Supplement: Supplementary file 1 [file cells-10-01606-s001.zip › cells-1267179-supplementary.pdf]

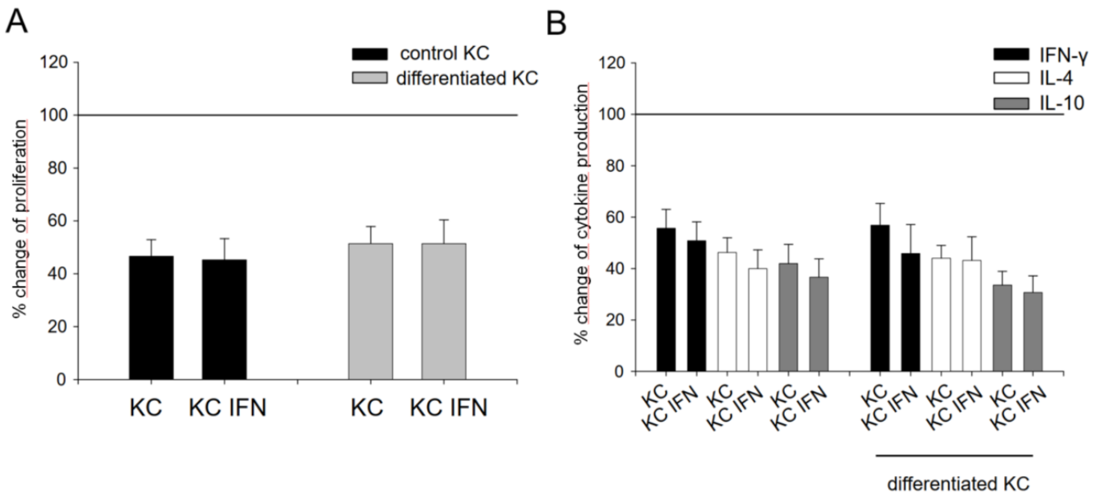

Figure S1. Keratinocyte differentiation does not impact the capacity of keratinocytes to inhibit T cell proliferation and cytokine production. *Keratinocytes (KC) were cultured under differentiating conditions (1.2mM CaCl2) for 24 hours before usage in co-culture experiments with T cells. After differentiation culture, KC or IFN-γ pre-stimulated KC (KC IFN), autologous nickel-specific T cell clones and DCs were incubated in a ratio of 10:100:1 in the presence of nickel-sulphate. After 48 hours, nickel-specific T cell proliferation was measured by 3H-Thymidine incorporation (A) and production of cytokines was detected in cell free co-culture supernatants by ELISA (B).*
